# Diurnal and Seasonal Variations of Photosynthetic Energy Conversion Efficiency of Field Grown Wheat

**DOI:** 10.3389/fpls.2022.817654

**Published:** 2022-02-25

**Authors:** Qingfeng Song, Jeroen Van Rie, Bart Den Boer, Alexander Galle, Honglong Zhao, Tiangen Chang, Zhonghu He, Xin-Guang Zhu

**Affiliations:** ^1^National Key Laboratory of Plant Molecular Genetics, CAS Center for Excellence in Molecular Plant Sciences, Institute of Plant Physiology and Ecology, Chinese Academy of Sciences, Shanghai, China; ^2^BASF Belgium Coordination Center – Innovation Center Gent, Ghent, Belgium; ^3^Institute of Crop Sciences, Chinese Academy of Agricultural Sciences, Beijing, China

**Keywords:** energy conversion efficiency, field crop, light use efficiency, radiation use efficiency, canopy chamber, canopy photosynthesis, CO_2_ flux, wheat

## Abstract

Improving canopy photosynthetic light use efficiency and energy conversion efficiency (ε_*c*_) is a major option to increase crop yield potential. However, so far, the diurnal and seasonal variations of canopy light use efficiency (LUE) and ε_*c*_ are largely unknown due to the lack of an efficient method to estimate ε_*c*_ in a high temporal resolution. Here we quantified the dynamic changes of crop canopy LUE and ε_*c*_ during a day and a growing season with the canopy gas exchange method. A response curve of whole-plant carbon dioxide (CO_2_) flux to incident photosynthetically active radiation (PAR) was further used to calculate ε_*c*_ and LUE at a high temporal resolution. Results show that the LUE of two wheat cultivars with different canopy architectures at five stages varies between 0.01 to about 0.05 mol CO_2_ mol^–1^ photon, with the LUE being higher under medium PAR. Throughout the growing season, the ε_*c*_ varies from 0.5 to 3.7% (11–80% of the maximal ε_*c*_ for C_3_ plants) with incident PAR identified as a major factor controlling variation of ε_*c*_. The estimated average ε_*c*_ from tillering to grain filling stages was about 2.17%, i.e., 47.2% of the theoretical maximal. The estimated season-averaged radiation use efficiency (RUE) was 1.5–1.7 g MJ^–1^, which was similar to the estimated RUE based on biomass harvesting. The large variations of LUE and ε_*c*_ imply a great opportunity to improve canopy photosynthesis for greater wheat biomass and yield potential.

## Introduction

Crop radiation use efficiency is the efficiency with which a crop utilizes absorbed light energy for biomass production and is calculated as the ratio of biomass accumulation per unit of absorbed or intercepted photosynthetically active radiation (PAR) (Monteith and Moss, 1977; Sinclair and Muchow, 1999; Hatfield, 2014). Increasing radiation use efficiency (RUE) is an important option to increase crop biomass production and yield potential (Reynolds et al., 2000; Zhu et al., 2010). Since biomass production is determined not only by activities of the source tissue but also by activities of the sink tissue, RUE is inherently determined by both source and sink activities. The correlations between biomass (or yield) and canopy photosynthesis (Wells et al., 1986) as well as leaf photosynthesis (Peng et al., 1991) were reported. Canopy photosynthesis is influenced by both the canopy architecture (Song et al., 2013; Burgess et al., 2017) and leaf photosynthetic capacities at different layers of the canopy (Murchie et al., 2002). Differences in canopy architecture can influence canopy microclimate, especially the light environments inside a canopy (Burgess et al., 2017). Many studies also show a strong influence of sink-related activities on biomass production and RUE (see review in Chang et al., 2017).

Radiation use efficiency (RUE) can be estimated based on either absorbed PAR or intercepted PAR. The absorbed PAR (APAR) is calculated as incident PAR less transmitted and reflected PAR (Lindquist et al., 2005; Slattery et al., 2017). The intercepted PAR (IPAR) is the incident PAR less transmitted PAR, which can be measured with PAR sensors (Ceotto and Castelli, 2002) or be predicted based on the canopy extinction coefficient (Hatfield, 2014) or with vegetation index, e.g., normalized difference vegetation index (NDVI), ratio vegetation index (RVI) and perpendicular vegetation index (PVI) (Hatfield, 2014). Furthermore, solar radiation instead of PAR has also been used to calculate RUE. The energy fraction of PAR is about half of the solar radiation and this relationship is used for the conversion between the PAR-based and solar radiation-based RUE (Sinclair and Muchow, 1999). Above-ground biomass rather than the total biomass is commonly used to estimate RUE because destructive sampling of above-ground biomass is much easier than harvesting the roots (Sinclair and Muchow, 1999). Studies of RUE with these methods show that there are large variations of RUE between different crops and under different treatments. Zhang et al. (2009) measured RUE in six different rice cultivars grown in different locations and found that RUE differs between cultivars, and also between plants grown at different locations for the same cultivar. For example, a rice cultivar Liangyoupeijiu had a RUE of 1.38 g MJ^–1^ in location Liuyang, while it was 1.52 in location Guidong. In Liuyang, the RUE of the rice cultivar II-you 838 was 1.26 g MJ^–1^, while the RUE for wheat cultivar Yangdao 6 was 1.45 g MJ^–1^, showing major differences between cultivars. Agronomic practice can also influence crop RUE. For example, in kenaf (*Hibiscus cannabinus* L.), reducing water and nitrogen soil availability lead to a decreased RUE (Li et al., 2008; Patanè and Cosentino, 2013); in wheat, furrow planting crops showed higher RUE compared to uniform planting, bed planting or wide-narrow row planting crops (Li et al., 2008). A meta-analysis based on 140 published studies further shows that environmental factors, such as elevated carbon dioxide (CO_2_), shade, intercropping, and nitrogen fertilizer application can also increase crop RUE, while other factors, such as elevated O_3_, water stress, foliar damage, and temperature stress, decrease crop RUE (Slattery et al., 2013).

Different from RUE, light use efficiency (LUE) represents the efficiency of plants to convert the absorbed light into gross or net CO_2_ uptake (Franklin, 2007). LUE can be measured within a short time, such as several minutes, and quantified for a day. LUE is defined as the net CO_2_ assimilation divided by absorbed PAR and the net CO_2_ assimilation equals photosynthesis minus plant respiration. The LUE can be estimated at either the leaf or canopy level (van Rooijen et al., 2015; Slattery et al., 2017). Canopy LUE is influenced by both leaf photosynthetic properties and canopy architecture. Many options to improve canopy photosynthesis rely on the modification of leaf photosynthetic properties (Long et al., 2015). Besides photosynthetic properties, canopy LUE is also influenced by leaf biochemical compositions. For example, canopy LUE is negatively correlated with canopy nitrogen use efficiency in *Abutilon theophrasti* and *Ambrosia artemisiifolia*, because more nitrogen investment to canopy will results in higher leaf area and canopy photosynthesis rate (Hirose and Bazzaz, 1998). Canopy architecture is another major factor controlling LUE since canopy architecture influences light environments inside a canopy. For example, peach tree canopies with the pyramid, parallelogram, or Y shape architectures (Giuliani et al., 2016) show different LUE; similarly, cotton canopies with different architectures as a result of changed growing densities also show major differences in LUE (Yao et al., 2017).

Besides these photosynthetic properties and canopy architectural parameters, canopy RUE and LUE are also influenced by environmental factors, such as light, CO_2_ levels, humidity, etc. As a result, there are large variations of RUE among different crops (Slattery and Ort, 2015). So far, most studies on RUE and LUE were conducted for a long experimental duration, i.e., these values were most measured on a weekly, monthly or seasonal basis (see review in Slattery and Ort, 2015). By comparison, the diurnal and seasonal variations of RUE and LUE for the same canopy are relatively less explored. In this study, we aim to characterize the variations of RUE and LUE in wheat canopies in the field and explore potential factors controlling these variations. Our measurements show that there is up to a fivefold difference in LUE for wheat canopies in the field, furthermore, wheat canopies can reach 11–80% of the maximal ε_*c*_ for C_3_ plants.

## Materials and Methods

### Plant Materials

In this study, two wheat cultivars with different plant architecture, namely Zhengyin 1 (ZY1) and Yumai 2 (YM2) (Supplementary Figure 1), were sown on 19 November 2016, in the Songjiang Experimental Station in Shanghai (N30.9*^o^*, E121.1*^o^*). The planting density was 100 kernels m^–2^. For each cultivar, 18 plots were used. Each plot included 7 rows with a row spacing of 0.2 m and a row length of 1.4 m. The center of each plot with an area of 1 m × 1 m was used for canopy photosynthesis measurements to avoid the border effect as described in Song and Zhu (2018). We followed the typical agricultural management practice in the Shanghai region. Fertilizers were applied before sowing following standard practice with 150 kg/ha N, 60 kg/ha P, and 30 kg/ha K.

### Multi-Chamber Canopy Photosynthesis and Transpiration System

**FIGURE 1:**
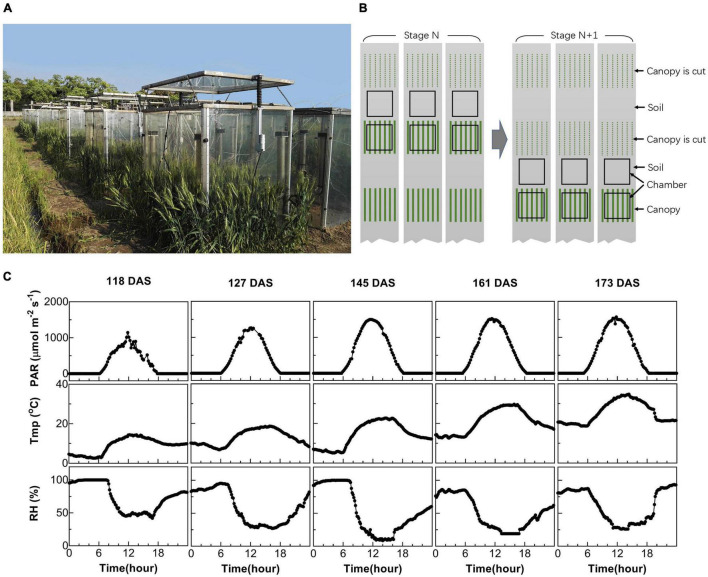
Measurement of canopy photosynthesis on field-grown wheat. **(A)** Multi-chamber canopy photosynthesis and transpiration system (multi-CAPTS) used for field canopy gas exchange measurement. **(B)** Diagram showing the logistics during the measurements. At stage N, three canopy chambers were used to measure the whole system’s CO_2_ flux, while three other chambers were used to measure the CO_2_ flux for soil. These canopy chambers were moved out of the field after the measurements at each stage; the plants used for the gas exchange measurement were harvested for biomass dry weight measurements. The chambers were then moved to new plots for gas exchange measurement at the next (N+1) stage. **(C)** Diurnal changes of environmental factors, including photosynthetic active radiation (PAR), air temperature (Tmp), and air relative humidity (RH) for 5 days at different stages when canopy photosynthesis were measured.

Two multi-chamber canopy photosynthesis and transpiration systems (multi-CAPTS) were built to determine canopy net CO_2_ assimilation rates during the daily photoperiod and canopy + soil respiration rates at night (Figure 1A). Each multi-CAPTS has one console that can be connected to 10 chambers and each chamber is the same as the previously reported single-chamber CAPTS (Song et al., 2016). The chamber wall is made of transparent polycarbonate (PC) film with a thickness of 1.5 mm. The transmittance of the chambers is 75.4% for PAR and the shade of the chamber frame was ignored. Only a small proportion of light was scattered by the film used in multi-CAPTS. Hence, the scattering by the film was ignored. To evaluate the effects of light scattering on canopy photosynthesis, we tested the effect of light scattering by covering the chamber with a scattering film that can convert 50% direct light to scattering light. Results show that the canopy photosynthesis can be increased up to 6.3% when the chamber was covered by such a scattering film (data shown in Supplementary Table 1). The frame of the chamber is made of a metal strip with a width of 30 mm, which can shade the canopy by about 3–6% at different time points during a day (a top view of the chamber and frame shadow was shown in Supplementary Figure 2). We also measured the transmittance of the PC film under different light incident angles (sun elevation angles) and the transmittance decreased by 4.4% when the incident angle changed from 0^°^ to 70^°^ (data shown in Supplementary Table 2). When the solar elevation is changing during a day, the change of chamber transmittance should be about 4.4% and the impact on the derived A_*cr*_-Q curve and the LUE estimation is limited. The chamber errors about the closed chamber system were reviewed by (Pérez-Priego et al., 2015).

The size of these chambers is 1 m × 1 m × 1.2 m (L × W × H). There were four fans installed at four corners of the chamber and the fans blowing air from top to the bottom for mixing air when the chamber was closed. When the chamber was open, the fans can also help to ventilate air between the inside and outside of the chamber because the fans were close to the top edge of the chamber. The air was ventilated between inside and outside of the chamber because the height of the chamber is higher than the height of the plant canopy, hence the chamber can block airflow between the canopy and outside air. The air ventilation can help to restore the CO_2_ and H_2_O concentrations in the chamber to the ambient levels. In the console, there is a multiplexer that can switch the gas from 1 of the 10 chambers to the infrared gas analyzer (IRGA). The console measures and records the CO_2_ concentration of the gas from a chamber with a 1 s interval when 1 of the 10 chambers is set to close automatically by the console. Plants are enclosed in the chamber, i.e., when the lid of the chamber is closed for 90 s for a single measurement. The temperature was increased by about 1*^o^*C inside the chamber during the 90 s closure. After a measurement is finished, the chamber is maintained open, and the next chamber is closed for measurement. The measurement starts from chamber 1 to 10 and then goes back to chamber one, sequentially. The console controlled the chambers’ opening and closing during the day. The data recorded by the console are used to calculate the rate of CO_2_ concentration change with time (*dc/dt*). The net CO_2_ flux (*F*_*c*_) including canopy, root, and soil flux, is calculated with Equation S1, as used in previous studies (Steduto et al., 2002).

In this study, we used six chambers associated with each console, with three chambers used to measure soil flux and three chambers used to measure the flux from the whole plant (canopy plus root) and soil together. The measurement layout of the chambers used in the field is shown in Figure 1B. We used six plots per genotype and per time point for the experiments. Among these, three wheat plots were used for the canopy photosynthesis and respiration measurements and harvesting above-ground biomass (BM) after the measurement. Three other plots where no plants were grown were used for the soil respiration measurements (Figure 1B). During the measurement of soil respiration, any weeds from the plots were manually removed. The canopy chambers remained on the plots for 2–3 days’ diurnal measurements at each developmental stage and then were removed to minimize the disturbance of the chamber on crop growth. At the next stage, the chambers were used to measure canopies in new plots (Figure 1B).

### Calculation of Net Plant CO_2_ Flux

The net CO_2_ flux for the whole system (*F*_*c*_) was determined with each chamber covering vegetation, which reflects the canopy gross photosynthesis, and respiration from canopy, root, and soil. In addition, the soil heterotrophic CO_2_ efflux rate (*R*_*h*_) was determined with a chamber covering soil free of vegetation and crop root tissue. The net plant CO_2_ flux (*A*_*cr*_) was calculated as the difference between *F*_*c*_ and *R*_*h*_ (the *R*_*h*_ is a negative value).


(1) $$A_{c\,r}=F_{c}-R_{h}$$

In Equation 1, the *A*_*cr*_ at night is the plant respiration rate (*R*_*cr*_).

### Calculation of Daily Net Plant CO_2_ Assimilation

During the day, the net CO_2_ flux for the whole system (*F*_*c*_) and the soil (*R*_*h*_) were measured at a fixed time interval, which was used to calculate the net plant CO_2_ flux at the particular time point (*A*^i^*_*cr*_*), with *i* being the number of the measurements, which ranges from *1* to *N*. The daily integral of net plant CO_2_ assimilation (*A*_*cr*,d_) was calculated as the product of the sum of *A*^i^*_*cr*_* with *i* from *1* to *N* and time interval from the *i^th^* to (*i+1*)*^th^* measurement (Equation 2). Similarly, the daily photosynthetically active radiation (*I*_*a,d*_) can be calculated with Equation 3.


(2) $$A_{\mathrm{cr},d}=\sum_{i=1}^{N}A_{\text{cr}}^{i}\times t$$

(3) $$I_{a,d}=\sum_{i=1}^{N}I_{\text{a}}^{i}\times t$$

where *N* is the number of measurement times in a day; *t* is the time interval between measurements; *I^i^*_*a*_ is the absorbed light at the *i^th^* measurement.

### Determination of Canopy Light Use Efficiency

The canopy LUE is calculated as the ratio between daily net whole plant CO_2_ assimilation (*A*_*cr,d*_) and the daily total canopy absorbed PAR per ground area (*I*_*a,d*_) (Equation 4).


(4) $$L\,U\,E=\frac{A_{c\,r,d}}{I_{a,d}}$$

### Fitting of Canopy Light Response Curve

The measured *A*_*cr*_ and *I* throughout a day can be used to reconstruct a canopy light response curve (A_*cr*_-Q curve, Q represent the Quantum flux density of incident light *I*), which can be fitted using a non-rectangular hyperbola curve (Equation 5). The canopy A_*cr*_-Q curve is an adaptation from a leaf light response curve (A-Q curve) (Johnson and Thornley, 1984). Note that the *A*_*cr*_ is the whole plant CO_2_ flux including both canopy and root. *R*_*cr*_ is the plant respiration including both canopy and root (positive value of *R*_*cr*_ was used in Equation 5). Curve fitting was performed with the *cftool* toolbox implemented in MATLAB software version R2020b (MathWorks, United States).


(5) $$A_{c\,r}=\frac{\Phi_{c}\cdot I+P_{c,m\,a\,x}-\sqrt{{\left(\Phi_{c}\cdot I+P_{c,m\,a\,x}\right)}^{2}-4\cdot \theta_{c}\cdot \Phi_{c}\cdot I\cdot P_{c,m\,a\,x}}}{2\cdot \theta_{c}}-R_{c\,r}$$

In this equation, the *P*_*c,max*_ is the maximal canopy photosynthetic CO_2_ uptake rate, Φ*_*c*_* is the quantum yield of canopy photosynthetic CO_2_ uptake, and *θ_*c*_* is the convexity factor (between 0 and 1) of the non-rectangular hyperbola describing the response of *A*_*cr*_ to *I*. For the leaf-level light response curve, the convexity factor is affected by the gradient of light absorption through the leaf and the photosynthetic capacity of the chloroplasts through the leaf. The convexity factor of chloroplast suspension is very close to 1 (Terashima and Saeki, 1985). The leaf light response curve is thought to be the sum of many individual light response curves (Ögren and Evans, 1993). Similarly, the canopy light response curve is the sum of light response curves of individual leaves in the canopy. Hence, the convexity factor derived from the A_*c*_-Q curve may similarly be influenced by the heterogeneity of the light environments and leaf photosynthetic properties inside the canopy. The *R*_*cr*_ is the rate of canopy plus root respiration. We used the measured *R*_*cr*_ at night in the fitting of the canopy light response curve.

### Calculation of Plant Net CO_2_ Flux for the Whole Growing Season

As the plant net CO_2_ flux (*A*_*cr*_) was only experimentally determined for a limited number of representative days, we predicted the *A*_*c*_ for the other days based on the A_*cr*_-Q curve (Equation 5) and the ambient PAR (*I*), which is continuously recorded with an interval of 10 min during the whole growing season using a weather station in the field. With the A_*cr*_-Q and *I*, the diurnal *A*_*cr*_ was calculated at a time interval of 10 min, and then *A*_*cr,d*_ was calculated as the integration of *A*_*cr*_ predicted at each time point during a day according to Equation 2.

### Estimation of Radiation Use Efficiency

Radiation use efficiency (RUE) is estimated based on two methods. For the first method, we directly calculated RUE as the slope of the linear regression between cumulative above ground BM (the method of biomass sampling and measurement was provided in the Supplementary File) over cumulative absorbed PAR. The canopy light absorption coefficient (α) was calculated based on the measurement of the incident, reflected, and transmitted PAR (see the Supplementary Method section). For the second method, we first calculated the cumulative biomass (predicted BM) from net CO_2_ uptake and then calculated the slope of the linear regression between predicted BM and corresponding canopy absorbed solar energy (E) for PAR (Equation 6):


(6) $$R\,U\,E=\frac{B\,M}{E}$$

To do this, we first calculated the potential biomass produced if all assimilated CO_2_ is stored in the form of carbohydrate (C_6_H_10_O_5_)_*n*_, such as cellulose and starch, where carbon atoms account for 44.4% of the total mass. Therefore, the theoretical biomass accumulation per day per unit ground area (BM_*d*_) can be calculated based on the net total canopy CO_2_ uptake rate (Equation 7), where the value of 12 (g mol^–1^) is the mole mass of carbon.


(7) $${\text{BM}}_{\text{d}}=\frac{A_{c\,r,d}\times 12\,g\,m\,o\,l^{-1}}{44.4\%}$$

In this study, the PAR was recorded with a weather station (WatchDog 2700, Spectrum Technologies Inc., Aurora, IL, United States). The total amount of energy absorbed by the canopy is calculated as:


(8) $$E=I_{a,d}\times 218\,k\,J\,m\,o\,l^{-1}$$

Where 218 (kJ mol^–1^) is the mean energy of one mol of a photon of the PAR (400–700 nm) in the solar light spectrum. This number was calculated using an Excel table in Supplemental Table 3. If we consider PAR in the range of 400–740 nm, this value is 205 (kJ mol^–1^) according to the previous calculation (Zhu et al., 2008).

### Calculation of Energy Conversion Coefficient From Solar Radiation to Biomass (ε_*c*_)

The energy conversion coefficient (ε_*c*_) is defined as the proportion of solar radiation that is converted into chemical energy and stored in biomass. The energy stored in biomass is assumed as 17.5 kJ g^–1^ (Monteith and Moss, 1977). The proportion of energy for PAR is 48.7% of total solar radiation (Zhu et al., 2008). The ε_*c*_ (unit: %) is calculated as Equation 9 and the conversion from RUE to ε_*c*_ can be calculated as Equation 10.


(9) $$\varepsilon_{c}=\frac{B\,M\times 17.5\,k\,J\,g^{-1}}{E/0.487}\times 100\%$$

(10) $$\varepsilon_{c}=R\,U\,E\times 0.487\times 17.5\,k\,J\,g^{-1}\times 100\%$$

Where 17.5 (kJ g^–1^) is the energy stored in biomass (Monteith and Moss, 1977) and 0.487 represents that 48.7% of the total solar radiation is PAR (Zhu et al., 2008).

### Statistics

The linear fitting was done with MatLab *cftool* package (MathWorks, United States; version R2020b), and the *ttest2* function in MatLab was used to perform the Student’s *t*-test. Statistical analysis of linear regression was done with *lm* function in RStudio version 1.4 (Boston, MA, United States).

## Results

### Diurnal Plant CO_2_ Flux, Light Use Efficiency, and Energy Conversion Efficiency of Wheat at Different Developmental Stages Measured Using Multi-CAPTS

**FIGURE 2:**
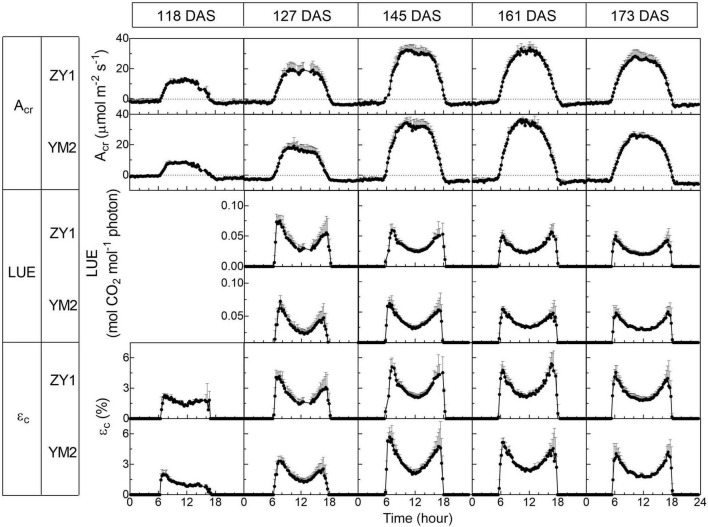
Diurnal changes of the whole plant (canopy plus root) CO_2_ flux (A_*cr*_), light use efficiency (LUE), and energy conversion efficiency (ε_*c*_) of two wheat cultivars, ZY1 and YM2, on different days at five developmental stages, i.e., the tillering (118 DAS), booting (127 DAS), heading (145 DAS), early grain filling (161 DAS), and late grain filling (173 DAS). The measurements were conducted with a multi-CAPTS system. Data are shown as mean ± *SD* (*n* = 3 plots).

**FIGURE 3:**
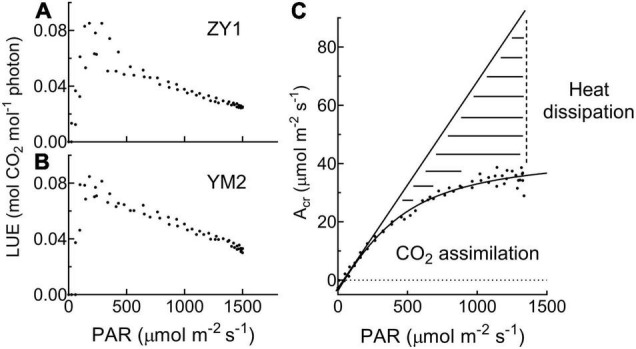
The relationship between light use efficiency (LUE) and photosynthetic active radiation (PAR) is based on data at the heading stage for ZY1 **(A)** and YM2 **(B)**. The response of whole plant CO_2_ flux (A_*cr*_) under different PAR **(C)** shows that a large portion of the incident light is used for heat dissipation, rather than photochemistry. The dashed area shows the light used for heat dissipation.

Diurnal canopy gas exchange of two wheat cultivars (ZY1 and YM2) with different plant architecture (Supplementary Figure 1) was measured using the multi-CAPTS (Figure 1A) at five different stages. The diurnal curves of PAR, air temperature (Tmp), and air relative humidity (RH) for the 5 days were recorded (Figure 1C). The stem height of ZY1 was higher (*p* < 0.01, Student’s *t*-test) than YM2 at the booting stage (Supplementary Figures 1A,C), but not significantly different at the early grain filling stage (Supplementary Figures 1B,C). The LAI of ZY1 was higher than YM2 at the booting stage (*p* < 0.01, Student’s *t*-test) and at the early grain filling stage (*p* < 0.1, Student’s *t*-test) (Supplementary Figure 1D). Diurnal variation of net plant CO_2_ flux (*A*_*cr*_) for different developmental stages was measured under the ambient incident PAR for ZY1 and YM2 (Figure 2). During the daytime from 06:00 a.m. to 6:00 p.m., the net *A*_*cr*_ was positive, i.e., the total canopy photosynthetic CO_2_ uptake rate was higher than the total rates of the canopy and root respiration. The maximal net *A*_*cr*_ at around 12:00 p.m. was the representative of diurnal whole plant CO_2_ uptake rate and *A*_*cr*_ on the 5 days was 13.5, 19.9, 32.4, 33.7, and 28.3 μmol m^–2^ ground s^–1^ for ZY1 and 8.5, 19.2, 34.6, 36.6, and 26.8 μmol m^–2^ ground s^–1^ for YM2. At night from 6:00 p.m. to 06:00 a.m. of the next day, the recorded *A*_*cr*_ was negative, reflecting the lack of photosynthesis during most of this period or the relatively low photosynthetic rate compared to total respiration rates from canopy and root (Figure 2). Furthermore, the diurnal LUE and energy conversion efficiency (ε_*c*_) were calculated based on the diurnal *A*_*cr*_ and PAR. The LUE and ε_*c*_ were lower under high light (at noon) than that under low light (early morning and late afternoon) (Figure 2). The LUE ranged from 0.02 to 0.075 mol CO_2_ mol^–1^ photon and the ε_*c*_ varied from 0 to 5.7% during the day at different developmental stages (Figure 2). To investigate the relationship between LUE and PAR, we plotted the relationship with the data at the heading stage for ZY1 and YM2. Results show that when PAR increased from zero to maximal, the LUE first increased and then decreased (Figures 3A,B). The response of whole plant CO_2_ flux (A_*cr*_) under different PAR was used to show that a large portion of the incident light is used for heat dissipation, rather than photochemistry (Figure 3C).

### Fitting of the Canopy Light Response Curves (A_*cr*_-Q Curve) at Different Stages

**FIGURE 4:**
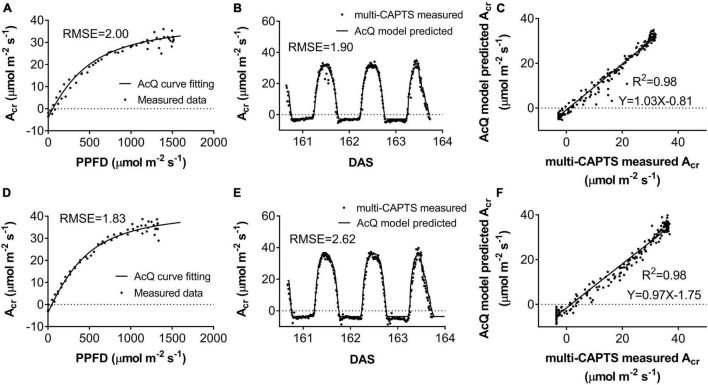
The response of whole plant CO_2_ flux (A_*cr*_) vs. photosynthetically active radiation (PAR) per ground area at early grain filling stage for field-grown wheat **(A,D)**. The comparison between predicted vs. measured A_*cr*_ **(B,E)**. The predicted (black curves) and measured (scatter points) diurnal changes of A_*cr*_ for ZY1(B) (RMSE = 1.9 μmol m^–2^ s^–1^) and YM2 (E) (RMSE = 2.62 μmol m^–2^ s^–1^). The predicted A_*cr*_ was based on recorded incident PAR and the parameters from the A_*cr*_-Q curve reconstructed with A_*cr*_ data and incident PAR on the 161st day after sowing (DAS). The predicted and measured A_*cr*_ were plotted against each other for ZY1 **(C)** and YM2 **(F)**. The R-square of linear regression is 0.98 for both ZY1 and YM2.

The diurnal variation of *A*_*cr*_ together with the diurnal variations of incident PAR enabled us to reconstruct an A_*cr*_-Q curve for a particular day (Figures 4A,D and Supplementary Figure 3). The R-squares of the curve fittings of the reconstructed A_*cr*_-Q curves using non-rectangular hyperbola were all higher than 0.9 for both cultivars at the five stages (Table 1), showing that the non-rectangular hyperbola curve, which is often used to represent the light response of leaf photosynthesis (Thornley, 2002), can also be used to effectively describe the response of canopy photosynthesis to light. Along with the progression of developmental stages, the maximal canopy photosynthetic CO_2_ uptake rate (*P*_*c,max*_) gradually increased from about 16.3 to 46.4 μmol m^–2^ ground s^–1^ for ZY1, and from about 9.9 to 48 μmol m^–2^ ground s^–1^ for YM2 (Table 1). Concurrently, the quantum yield of canopy photosynthetic CO_2_ uptake (Φ*_*c*_*) increased from the tillering stage (0.034 mol CO_2_ mol^–1^ photon for ZY1 and 0.02 mol CO_2_ mol^–1^ photon for YM2) to the heading stage (0.091 mol CO_2_ mol^–1^ photon for ZY1, 0.09 mol CO_2_ mol^–1^ photon for YM2), and then decreased at the early grain filling stage (0.075 mol CO_2_ mol^–1^ photon for ZY1, 0.082 mol CO_2_ mol^–1^ photon for YM2) (Table 1). The A_*cr*_-Q curve was further used to predict the A_*cr*_ of all days during the growing season. The accuracy of this method to estimate canopy photosynthesis was tested by comparing the measured *A*_*cr*_ using the CAPTS for 1 day and the predicted *A*_*cr*_ for that same day using an A_*cr*_-Q curve obtained on other recent days (Figures 4B,C,E,F and Supplementary Figure 4). The R-square between *A*_*cr*_ derived from these two approaches varies between 0.784 (Supplementary Figure 4D) and 0.98 (Figure 4C) (e.g., ZY1: R^2^ = 0.98 and RMSE = 1.9 μmol m^–2^ s^–1^, YM2: R^2^ = 0.98 and RMSE = 2.62 μmol m^–2^ s^–1^, Figures 4B,C,E,F). The accuracy of the A_*cr*_-Q curve model prediction is influenced by the diurnal variations of temperature and humidity, which can influence leaf CO_2_ fixation capacity (Bernacchi et al., 2001) and stomatal conductance (Ball et al., 1987). We used data from different weather to build the A_*cr*_-Q model and predicted the A_*cr*_ of the days at the same stage and the RMSE was calculated to evaluate the accuracy of model prediction (Supplementary Table 4). Estimate *A*_*cr*_ on a cloudy day using A_*cr*_-Q parameters from the sunny day has larger RMSE than that estimating *A*_*cr*_ on a sunny day. To further evaluate the impact of these parameters on the estimation of LUE and ε_*c*_, we conducted a sensitivity analysis on the influence of altering *P*_*cmax*_, Φ*_*c*_*, and *θ_*c*_* on the estimation of LUE (Supplementary Table 5) and ε_*c*_ (Supplementary Table 6). The A_*cr*_-Q model used in the current study did not include the temperature effect. To evaluate the temperature effect to the photosynthetic parameters including *P*_*cmax*_, Φ*_*c*_*, and *θ_*c*_*, we conducted linear regression analysis for each of these parameters with independent variables including temperature, stage, cultivar, and replicate. Statistical analysis showed that temperature significantly influenced *P*_*cmax*_ and Φ*_*c*_*, but not *θ_*c*_* (Table 2). The growth stage was a major factor influencing *P*_*cmax*_, Φ*_*c*_*, and *θ_*c*_* as plant canopies at different stages were dramatically different for both leaf area and photosynthetic capacity. We further did the analysis for data from each stage respectively, the temperature effect to *P*_*cmax*_, Φ*_*c*_*, and *θ_*c*_* was not significant in most situations except at the early grain filling stage when the temperature effect to Φ*_*c*_* was significant (Table 2). To estimate the season-long *A*_*cr*_, we divided the whole growing season into five different periods (Table 3), then we calculated the instantaneous *A*_*cr*_ throughout each period using the fitted *P*_*c,max*_, Φ*_*c*,_* and *θ_c_* based on the A_*cr*_-Q curve for 1 day in that period (Supplementary Figure 3) and the instantaneous incident diurnal PAR (*I*) recorded for each day in that period (Supplementary Figure 5).

**TABLE 1:** Parameters derived from A_*cr*_-Q curves, which were constructed from parallel measurements of whole-plant carbon dioxide (CO_2_) flux and of incident photosynthetically active radiation (PAR).

Cultivar	DAS	*P*_*cmax*_ (μmol m^–2^ s^–1^)	*Φ_*c*_* (μmol μmol^–1^)	*θ_*c*_*	*R*_*cr*_ (μmol m^–2^ s^–1^)	R-square	SSE	RMSE
ZY1	118	16.3 ± 0.6	0.034 ± 0.003	0.90 ± 0.02	2.1 ± 1.4	0.94 ± 0.01	75 ± 20	1.3 ± 0.2
	127	23.3 ± 6.3	0.050 ± 0.011	0.87 ± 0.04	2.8 ± 0.8	0.96 ± 0.02	112 ± 54	1.5 ± 0.4
	145	38.9 ± 5.2	0.091 ± 0.015	0.60 ± 0.20	2.9 ± 0.7	0.95 ± 0.03	367 ± 302	2.4 ± 1.0
	161	46.4 ± 8.6	0.075 ± 0.001	0.36 ± 0.31	2.9 ± 0.9	0.97 ± 0.02	264 ± 179	2.1 ± 0.7
	173	36.7 ± 7.8	0.079 ± 0.022	0.44 ± 0.06	3.2 ± 0.3	0.98 ± 0.01	118 ± 74	1.4 ± 0.4
YM2	118	9.9 ± 0.6	0.020 ± 0.002	0.97 ± 0.01	1.2 ± 0.3	0.91 ± 0.02	86 ± 26	1.2 ± 0.2
	127	20.9 ± 4.9	0.047 ± 0.009	0.92 ± 0.06	3.1 ± 0.5	0.95 ± 0.04	153 ± 59	1.6 ± 0.3
	145	40.6 ± 5.7	0.090 ± 0.017	0.74 ± 0.15	3.4 ± 0.9	0.96 ± 0.02	313 ± 120	2.3 ± 0.5
	161	48.0 ± 4.1	0.082 ± 0.016	0.48 ± 0.31	3.8 ± 1.0	0.96 ± 0.02	345 ± 217	2.4 ± 0.8
	173	38.8 ± 2.2	0.084 ± 0.005	0.13 ± 0.23	4.5 ± 0.5	0.97 ± 0.01	145 ± 35	1.6 ± 0.2

*The parameters derived from the A_cr_-Q curve were reconstructed from recorded A_cr_ using CAPTS and the concurrent recording of incident PAR.*

*P_cmax_, maximal canopy photosynthetic CO_2_ uptake rate; Φ_c_, quantum yield of canopy photosynthetic CO_2_ uptake; θ_c_, convexity factor of the A_cr_-Q curve; R_cr_, canopy and root respiration; SSE, sum of standard error; RMSE, root mean square error.*

*Two cultivars ZY1 and YM2 at 5 days during the growing season, 118th, 127th, 145th, 161st, and 173rd days after sow (DAS) were used for the curve fitting.*

**TABLE 2:** Statistical analysis of the significance of factors controlling canopy photosynthetic parameters (*P*_*cmax*_, *Φ_*c*_*, *θ_*c*_*) derived from light response curve of canopy photosynthesis (A_*cr*_-Q).

Dataset	Photosynthetic parameters	Main effects			
		Stage	Temp	Cultivar	Stage × Temp
All stages	*P*_*cmax*_	**1.42E-09**	**0.0015**	0.857	**4.79E-06**
	*Φ_*c*_*	**5.58E-07**	0.0143	0.928	**4.14E-04**
	*θ_*c*_*	0.989	0.074	0.234	0.089
Stage 1	*P*_*cmax*_	–	0.876	0.746	–
	*Φ_*c*_*	–	0.627	0.546	–
	*θ_*c*_*	–	0.467	0.498	–
Stage 2	*P*_*cmax*_	–	0.505	0.453	–
	*Φ_*c*_*	–	0.725	0.233	–
	*θ_*c*_*	–	0.593	0.573	–
Stage 3	*P*_*cmax*_	–	0.082	0.023	–
	*Φ_*c*_*	–	0.019	0.220	–
	*θ_*c*_*	–	0.186	0.661	–
Stage 4	*P*_*cmax*_	–	0.835	0.523	–
	*Φ_*c*_*	–	**7.83E-04**	**8.97E-04**	–
	*θ_*c*_*	–	**0.008**	0.059	–
Stage 5	*P*_*cmax*_	–	0.346	0.529	–
	*Φ_*c*_*	–	0.111	0.054	–
	*θ_*c*_*	–	0.587	0.171	–

*Datasets from all stages or each stage were used for the analysis. Stage, air temperature (Temp), cultivar (ZY1 and YM2) (n = 3 plots), and the interaction between stage and temperature (Stage × Temp) were used as main effects influencing P_cmax_, Φ_c,_ and θ_c_. Bold values represent the significance of p value < 0.01.*

**TABLE 3:** Overview of the dates of the five developmental stages on which canopy photosynthesis (A_*cr*_) was measured.

Developmental stage	The day used for A_*c*_ measurement (DAS)	Days covered by the A_*cr*_-Q model (Date)	Days covered by the A_*cr*_-Q model (Days after sowing, DAS)
Tillering	118	14 March–20 March	115–121
Booting	127	21 March–3 April	122–135
Heading	145	4 April–21 April	136–153
Early grain filling	161	22 April–6 May	154–168
Late grain filling	173	7 May–11 May	169–173

*The name of the developmental stage that each of these 5 days belongs to is shown. For every day in a particular developmental stage, the A_cr_ was calculated based on parameters derived from the A_cr_-Q curve measured for the day representing this developmental stage.*

### Daily Light Use Efficiency of Two Wheat Cultivars Along the Growing Season

**FIGURE 5:**
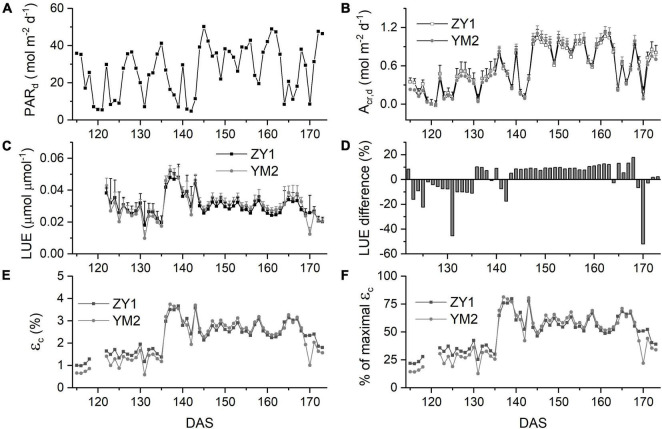
Canopy photosynthesis properties for two field-grown wheat cultivars. Daily total photosynthetically active radiation (PAR_*d*_) **(A)**, daily whole plant CO_2_ uptake rate (A_*cr*,d_) **(B)**, and light use efficiency (LUE) **(C)** of ZY1 and YM2 for each day from the tillering stage to the late grain filling stage. The difference of LUE between ZY1 and YM2, calculated as (YM2-ZY1)/ZY1*100%, is shown in panel **(D)**. Energy conversion efficiency (ε_*c*_), i.e., calculated as the energy stored in biomass over total incident solar radiation for each day is shown in panel **(E)**. The percentage of ε_*c*_ for two cultivars to the maximal ε_*c*_ was shown in panel **(F)**. Data in panels **(C,D)** are shown as mean ± *SD* (*n* = 3 plots).

**FIGURE 6:**
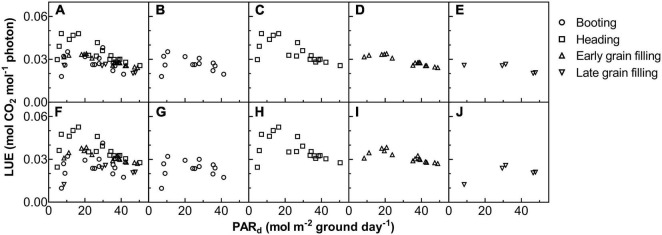
The relationship between LUE on daily total photosynthetically active radiation (PAR_*d*_) for ZY1 **(A–E)** and YM2 **(F–J)**. The shape of data points denotes the different growth stages. The data of all the four stages were shown together **(A,F)** and shown separately for booting **(B,G)**, heading **(C,H)**, early grain filling **(D,I)**, and late grain filling **(E,J)** stages.

**FIGURE 7:**
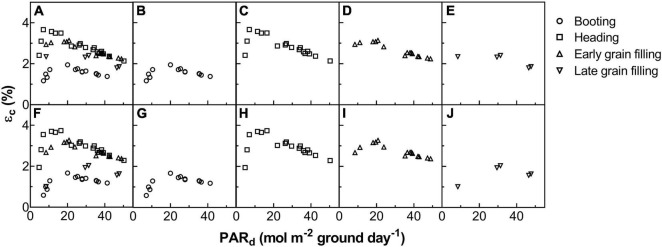
The relationship between energy conversion efficiency (ε_*c*_) and daily total photosynthetically active radiation (PAR_*d*_) for ZY1 **(A–E)** and YM2 **(F–J)**. The shape of data points represents different developmental stages. The data of all the four stages were shown together **(A,F)** and shown separately for booting **(B,G)**, heading **(C,H)**, early grain filling **(D,I)**, and late grain filling **(E,J)** stages.

The *A*_*cr,d,*_ and LUE for each day throughout the growing season were calculated (Figure 5). The *A*_*cr,d*_ varied dramatically between different days, mainly caused by variation of the daily PAR (PAR_*d*_) (Figures 5A,B). We also found dramatic changes in the LUE for different days (Figure 5C). The LUE of YM2 at the tillering and booting stages was lower than ZY1 while for most days at the grain filling stage, the LUE of YM2 was higher than ZY1 (Figure 5D). Energy conversion coefficient (ε_*c*_) for all these days was also calculated as the energy stored in biomass over total incident solar radiation (Figure 5E). ε_*c*_ varied from 0.5 to 3.7% during the growing season, i.e., about 11–80% of theoretical maximal ε_*c*_ of C_3_ plants (Figure 5F). The theoretical maximal ε_*c*_ of C_3_ plants has been calculated earlier (Zhu et al., 2008). We also calculated the average ε_*c*_ from tillering to the late grain filling stage (from the 115th to the 173rd DAS). Average ε_*c*_ was 2.19% for ZY1 and 2.16% for YM2 and they were 47.6 and 47% of theoretical maximal for the period from the 115th to the 173th DAS (ε_*c*_ 2.17 and 47.2% of theoretical maximal for the average of the two cultivars). If we calculate average ε_*c*_ from the day of sowing to 173th DAS, the average ε_*c*_ was 1% for ZY1 and 0.99% for YM2 and they were 21.7 and 21.4% of the theoretical maximal value. The relationship between LUE and PAR_*d*_ at different developmental stages is shown in Figure 6. Similarly, the relationship between ε_*c*_ and PAR_*d*_ at different stages were shown in Figure 7. When PAR_*d*_ increased, the LUE and ε_*c*_ initially increased and then decreased at all the stages (Figures 6, 7). The maximal LUE and ε_*c*_ occurred under medium PAR_*d*_ between 10 and 30 mol m^–2^ ground day^–1^ at different stages (Figures 6, 7).

### Biomass Accumulation and Season Average Radiation Use Efficiency

**FIGURE 8:**
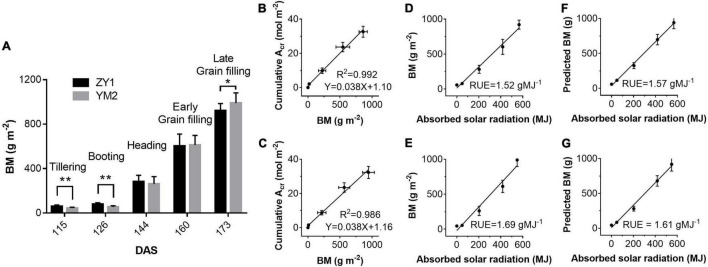
Above-ground biomass (BM) harvested on days at different stages **(A)** and the correlations between BM and cumulative plant CO_2_ uptake rate (A_*cr*_) for ZY1 **(B)** and YM2 **(C)**, respectively. Relationships between measured BM and absorbed solar radiation (equals to canopy absorbed solar energy for PAR divided by 0.487) for ZY1 **(D)** and YM2 **(E)** and the relationship between predicted biomass (predicted BM) and absorbed solar radiation for ZY1 **(F)** and YM2 **(G)** were plotted. The predicted BM was calculated from net whole plant CO_2_ flux measured with multi-CAPTS. RUE is the slope of linear regression of the BM vs. absorbed solar radiation and the predicted BM vs. absorbed solar radiation. Data in panel **(A)** are shown with mean ± *SD* (*n* = 15) and double asterisks show *p* < 0.005 and single asterisks shows *p* < 0.05 for Students’ *t*-test. Data in panels **(B–G)** are shown with mean ± *SD* (*n* = 3 plots).

Above ground BM of ZY1 and YM2 at different developmental stages was collected and the dry weight was measured to estimate RUE using the biomass-based approach. The dry weight of BM of ZY1 was significantly higher than YM2 at the tillering stage, i.e., represented by the 115th DAS, and the booting stage, i.e., represented by the 126th DAS (*P* < 0.01, *n* = 15) (Figure 8A), while at the late grain filling stage, i.e., the 173rd DAS, the BM of ZY1 was significantly lower than YM2 (*P* < 0.1, *n* = 15) (Figure 8A). We further analyzed the correlation between cumulative net plant CO_2_ assimilation (*A*_*cr*_) and BM. *A*_*cr*_ is highly linearly related to biomass accumulation (R^2^ = 0.992 for ZY1 and R^2^ = 0.986 for YM2) (Figures 8B,C). The RUE, estimated based on canopy absorbed solar radiation and above-ground biomass, was 1.52 g MJ^–1^ for ZY1 and 1.69 g MJ^–1^ for YM2 (Figures 8D,E). The RUE based on the predicted BM from cumulative *A*_*cr*_ was 1.57 g MJ^–1^ for ZY1 and 1.61 g MJ^–1^ for YM2 (Figures 8F,G). The average RUE of the two cultivars was 1.6 g MJ^–1^.

## Discussion

### Large Variations in Light Use Efficiency and ε_*c*_ in the Field and Their Contributing Factors

This article shows that in the field there are large diurnal and seasonal variations of canopy LUE, RUE, and ε_*c*_ estimated with chamber-based canopy photosynthesis measurements and recordings of diurnal changes of photosynthetic active radiation. The conventional method to estimate these efficiencies (Monteith and Moss, 1977; Sinclair and Muchow, 1999; Hatfield, 2014) is based on the measured biomass increment during a relatively longer period. Such methods, however, suffer from the large variations in biomass between plots caused by heterogeneities of field conditions, requirements for longer measurement intervals to enable accurate quantification of biomass increment, and the difficulty to accurately measure below-ground biomass (Sinclair and Horie, 1989; Lindquist et al., 2005). The method reported here enables estimation of LUE and ε_*c*_ at a much higher temporal resolution since the CO_2_ uptake rate can be measured at a time resolution of a minute.

With this new approach, we estimated the dynamic diurnal and seasonal changes of LUE and ε_*c*_. The large variations of LUE can be partially attributed to the variations in ambient light levels (Figures 3A,B). Under high light, the LUE is lower because more light energy was dissipated as heat and fluorescence (Figure 3C). This light-dependent LUE variation is consistent with the result of meta-analysis which shows that the energy conversion efficiency is higher under (partial) shading treatment (Slattery et al., 2013). The quality of light is another factor influencing LUE. In our experiment, the calculated LUE and ε_*c*_ were much higher on cloudy days than those under sunny days (Figure 5E). These results were consistent with earlier studies which show that more diffuse light on cloudy days may lead to higher LUE (Sinclair et al., 1992; Healey et al., 1998; Choudhury, 2000). Under diffuse light, canopy photosynthesis can be increased by redistributing light from light-saturated (sunlit) leaves to non-saturated (shade) leaves (Knohl and Baldocchi, 2008). The large variation of LUE might also be attributed to the large dynamic variations of PAR during a day (Figure 1 and Supplementary Figure 5) and during a season (Figure 5A) as well. Besides, the dynamic change of LUE during a season was also influenced by developmental stage and temperature (Figure 6 and Table 2). Many recent studies show that there is large scope to improve the efficiency of dynamic photosynthesis through the increase of either the speed of stomatal movement or the rate of recovery from the photoprotective state after plants change from high to low light (Lawson and Blatt, 2014; Kromdijk et al., 2016; Qu et al., 2020; Wang et al., 2020). Since the LUE can be measured at a time resolution of minute, the new method presented here can also be used to study the efficiency of dynamic photosynthesis in the field, which is becoming a major area of research in recent years (Kaiser et al., 2015; Wang et al., 2021).

Our data further show a large variation of LUE at different developmental stages and further suggest factors underlying these changes. At early developmental stages, ZY1 had a higher leaf area index than YM2 (Supplementary Figures 1A,C,D) and also a higher A_*cr*,d_ (Figure 5B); furthermore, A_*cr*,d_ during the sunny days gradually increased from tillering till the end of booting stages (Figure 5B), which were accompanied by a concurrent increase in canopy size. These suggest that the increase of leaf area index at early developmental stages is a major factor controlling LUE, which supports the selection of rapid development of canopy size as an important trait in crop breeding (Shi et al., 2020; Aharon et al., 2021). After canopy closure, canopy LUE depends not only on organ level photosynthetic efficiency, which includes photosynthesis of both foliar tissues (Murchie et al., 2002) and non-foliar tissues (Chang et al., 2020), but also on canopy architecture, such as leaf angle, leaf area index, etc. During the grain filling season, factors influencing LUE may include the duration of grain filling and the photosynthetic capacities of the canopy. In this study, from the 161st to the 173rd DAS, A_*cr*,d_ on the sunny days significantly decreased for both cultivars (Figure 5B), which can be attributed to the decrease of leaf photosynthetic capacity (Supplementary Figure 6). In this study, at the late grain filling stage, the leaf photosynthetic activity of ZY1 was lower than YM2 (Supplementary Figures 6D,H), but the leaf area of ZY1 was higher than YM2 (Supplementary Figure 1D) and the canopy absorbance of ZY1 was also higher than YM2 (Supplementary Figure 7). The *A*_*cr,d*_ of ZY1 was higher than YM2 (Figure 5D), showing that the higher LAI and corresponding higher canopy absorbance were the major factors contributing to the higher canopy photosynthesis in ZY1. In our earlier study, the capacity to maintain photosynthetic efficiency at later developmental stages is shown as a major contributing factor for higher biomass and yield formation in Huanghuazhan, an elite rice cultivar in China (Shi et al., 2019). Therefore, the canopy LUE and ε_*c*_ estimated at a high temporal resolution, when combined with the measured plant physiological parameters, plant architectural parameters, and environmental parameters, offer a new opportunity to study factors influencing LUE.

### Variations of the Light Response Curves of Canopy Photosynthesis at Different Developmental Stages

In this study, we used a non-rectangular hyperbola curve, which is widely used to model leaf light response of photosynthesis (Stirling et al., 1994) and to model the light response of canopy photosynthesis (A_*cr*_-Q) (Figure 4 and Table 1). Here, we show that there are large variations of *P*_*c,max*_, Φ_*c*,_ and *θ_*c*_* at different developmental stages (Table 1). The *P*_*c*_,_*max*,_ and Φ*_*c*_* showed a gradual increase from the tillering stage to the early grain filling stage. The gradual increase in *P*_*c*,*max*_ reflects the increase in canopy size and increased photosynthetic capacity along with wheat development. The Φ*_*c*_* is the maximal quantum yield of canopy photosynthesis, i.e., LUE under low light conditions. Considering that during a day, the incident PAR varies constantly, the much lower LUE estimated on different days compared to Φ*_*c*_* suggests that, during most times of the day, a large portion of the incident light energy is dissipated as heat and fluorescence, rather than used for photochemistry (Figure 3C), i.e., some leaves in the canopy receives more light than needed by photosynthesis. Therefore, there is still a scope to improve the canopy photosynthesis of these two cultivars through either optimizing architecture for improved light distribution inside the canopy or improving the carbon fixation capacity, which is not dependent on plant morphology.

We further found that, after a canopy is closed, even when the incident PAR reached around 1,500 μmol m^–2^ s^–1^, the A_*cr*_-Q still did not reach a plateau, which is drastically different from a light response curve of a leaf, typically showing a light saturation around 1,200 μmol m^–2^ s^–1^ (Supplementary Figures 6B–D). The continuing increase in A_*cr*_ after 1,500 μmol m^–2^ s^–1^ suggests that the canopy architecture of these two cultivars can enable a relatively even distribution of light inside the canopy, in contrast to a scenario where most of the light is intercepted by top-layer leaves. The fitted *θ_*c*_* showed a value varying in most cases between 0.4 and 0.9 (Table 1). Earlier, the θ derived from the light response curve of chloroplast suspension has been shown to be around 1 (Terashima and Saeki, 1985), suggesting that θ is related to the heterogeneity of light inside a leaf. Xiao et al. (2016) showed that the variation of θ derived from light response curves of leaf photosynthetic CO_2_ uptake rate reflects the heterogeneities of not only the microclimate but also the photosynthetic capacity inside a leaf. In this case, the much lower *θ_*c*_* observed at grain filling stages for two cultivars compared to those at earlier stages (Table 1) suggests that both the heterogeneities of light environments and photosynthetic properties inside the canopies differ between developmental stages. Furthermore, our analysis shows that ZY1 had a higher *θ_*c*_* than YM2 (Table 1), which again can be attributed to the different microclimate and photosynthetic properties in canopies for these two cultivars. The leaves of YM2 were mainly erect while the leaves of ZY1 were mostly horizontal (Supplementary Figure 1B); this difference in plant architecture influenced the light environments in a canopy. Canopy absorbance measurement confirmed that more light penetrated to the bottom layer of YM2 (Supplementary Figure 7). Furthermore, the leaf photosynthetic capacity (*A*) was also different between the two cultivars. In YM2, *A* of the second leaf was similar to the flag leaf (Supplementary Figures 6C,D), showing that both the flag leaf and the second leaf contributed to the canopy photosynthesis. However, in ZY1, the *A* of the second leaf was dramatically lower than the flag leaf. Both the light environment and also the photosynthetic properties suggest that the canopy photosynthesis of ZY1 was mainly contributed by the flag leaf at the top layer of the canopy while for YM2 both the flag and the second leaves contribute significantly to total canopy photosynthesis (Supplementary Figures 6C,D), which may underlie the observed lower *θ_*c*_* in YM2, as in the case of chloroplast suspension as compared to a leaf (Xiao et al., 2016).

The A_*cr*_-Q curve enables the multi-CAPTS data to be used for the estimation of diurnal and seasonal variations of LUE and ε_*c*_. The estimated *P*_*c*_,_*max*_, Φ*_*c*_*, and *θ_*c*_* based on the multi-CAPTS data together with the recorded photosynthetic active radiation can be used to estimate canopy photosynthesis at a high temporal resolution. In theory, the accuracy of the A_*cr*_-Q model prediction depends on the temperature and humidity, since temperature and humidity influence photosynthetic parameters (Bernacchi et al., 2001) and stomatal conductance (Ball et al., 1987). We indeed notice that the difference between model predicted A_*cr*_ and measured A_*cr*_ on DAS 163 was larger because the temperature on the 163rd day was lower than that of the DAS 161. This suggests that the ability of this new approach to estimate LUE for other crops and under more diverse conditions needs to be systematically tested and methods to use the A_*cr*_-Q curve to predict A_*cr*_ on days with different temperatures and humidity need to be developed.

### Comparison of Chamber-Based Method and Biomass-Based Method to Estimate Radiation Use Efficiency

The whole plant CO_2_ flux (*A*_*cr*_) was calculated as the difference between the net CO_2_ flux for the plots with both plant and soil (*F*_*c*_), and soil respiration for the plots without plants (*R*_*h*_, heterotrophic respiration), which were directly measured with the multi-CAPTS in this study. The soil water contents for the plots without plants were different from those of the plots with crops because water uptake by root would be different. As the *R*_*h*_ is influenced by soil water content, the respiration/water content dependency function needs to be used to correct the *R*_*h*_ according to the measured soil water content following Prolingheuer et al. (2014). To compare the chamber-based and biomass-based methods, we predicted biomass dry weight from whole plant CO_2_ flux data and used it to estimate the season average RUE. To do this, we assume all biomass is stored in the form of carbohydrates, i.e., we ignore that a substantial fraction of biomass is in the form of proteins, lipids, and minerals (Murphy and Parker, 1984). Though with this simplification, we still found a strong positive correlation between the above-ground biomass and accumulated *A*_*cr*_ (Figures 8B,C), which justifies the usage of calculated BM to estimate the RUE. For comparative studies on RUE between cultivars or crops, measurements of the tissue biochemical compositions are necessary.

The estimated RUE for ZY1 with the biomass-based approach and the multi-CAPTS-based approach were 1.52 and 1.57 g MJ^–1^ (Figures 8D,F), respectively; the estimated RUE for YM2 with biomass-based and multi-CAPTS-based approaches were 1.69 and 1.61 g MJ^–1^, respectively (Figures 8E,G). These estimated RUE values were similar to the published values for wheat under non-stress conditions (Sinclair and Muchow, 1999; Li et al., 2008). Given that one mole of CH_2_O contains 477 kJ energy, a RUE of 1 g MJ^–1^ is equivalent to an energy conversion efficiency (ε_*c*_) of 1.6%. Based on these, the average ε_*c*_ from tillering stage to grain filling stage for these two wheat cultivars were estimated to be about 2.19% (ZY1) and 2.16% (YM2), i.e., the 47.6 and 47% of the theoretical maximum for C_3_ crops. The theoretical maximum ε_*c*_ for C_3_ crops was calculated by Zhu et al. (2008). It should be noted that if we calculate the average ε_*c*_ from the day of sowing to the late grain filling stage (173rd DAS), the average ε_*c*_ was 1% for ZY1 and 0.99% for YM2 and they were 21.7 and 21.4% of the theoretical maximal value. It is worth emphasizing here that when incident daily total PAR was between 10 and 30 mol m^–2^ ground day^–1^ (daily average PAR from 230 to 690 μmol m^–2^ s^–1^), the instantaneous ε_*c*_ at heading and grain filling stage reached 2.8–3.7% (Figure 6), which was 61–80% of the theoretical maximum.

## Data Availability Statement

The raw data supporting the conclusions of this article will be made available by the authors, without undue reservation.

## Author Contributions

X-GZ and JV conceived the project. QS analyzed the data and interpreted the results, as well as performed the field experiments with help from HZ, TC, and ZH. QS wrote the article with input from all authors. X-GZ, JV, BD, and AG revised the article. All authors contributed to the article and approved the submitted version.

## Conflict of Interest

The authors declare that the research was conducted in the absence of any commercial or financial relationships that could be construed as a potential conflict of interest.

## Publisher’s Note

All claims expressed in this article are solely those of the authors and do not necessarily represent those of their affiliated organizations, or those of the publisher, the editors and the reviewers. Any product that may be evaluated in this article, or claim that may be made by its manufacturer, is not guaranteed or endorsed by the publisher.

## Figures and Tables

**Table:** 

Symbol	Definition	Unit
*A*_cr_	Whole plant (canopy plus root) CO_2_ flux (CO_2_ uptake rate)	μmol CO_2_ m^–2^ ground s^–1^
*A*_cr, d_	Daily whole plant (canopy plus root) CO_2_ uptake rate	mol CO_2_ m^–2^ day^–1^
*A*^i^*_cr_*	Net plant CO_2_ flux at the particular time point *i*	μmol CO_2_ m^–2^ ground s^–1^
BM	Total biomass (dry weight)	g m^–2^ ground
BM_*d*_	Daily biomass accumulation (dry weight)	g m^–2^ ground day^–1^
*E*	Daily total incident light energy	MJ day^–1^
*F*_c_	Net CO_2_ flux of plant canopy, root and soil	μmol CO_2_ m^–2^ ground s^–1^
*I*	Incident PAR per ground area above a canopy	μmol m^–2^ ground s^–1^
*I*_*a*_	Absorbed PAR per ground area by a canopy	μmol m^–2^ ground s^–1^
*I*_*r*_	PAR per ground area reflected by canopy	μmol m^–2^ ground s^–1^
*I*_*t*_	PAR per ground area transmitted through canopy	μmol m^–2^ ground s^–1^
*I*_*a,d*_	Daily photosynthetically active radiation	μmol m^–2^ ground day^–1^
*I^i^*_*a*_	Absorbed light at the *i*^th^ measurement	μmol m^–2^ ground s^–1^
LUE	Net CO_2_ uptake per unit absorbed PAR by a canopy	mol CO_2_ mol^–1^ photon
PAR	Photosynthetically active radiation from 400 to 700 nm wavelength	μmol m^–2^ ground s^–1^
PAR_d_	Daily total photosynthetically active radiation	mol m^–2^ ground day^–1^
*P*_*c*,_ *_*max*_*	Maximal gross canopy photosynthetic CO_2_ uptake rate	μmol m^–2^ ground s^–1^
*R*_*cr*_	The whole plant (canopy plus root) respiration rate on a unit ground area basis	μmol m^–2^ ground s^–1^
*R*_h_	The soil heterotrophic respiration rate on a unit ground area basis	μmol m^–2^ ground s^–1^
RUE	Radiation Use Efficiency, calculated as the ratio of biomass production over absorbed solar radiation	g MJ^–1^
α	The canopy absorption coefficient of PAR, the ratio of absorbed PAR to incident PAR	Dimensionless
ε_c_	Canopy photosynthetic energy conversion efficiency	%
Φ_c_	The quantum yield of canopy photosynthetic CO_2_ uptake, which is the initial slope of canopy photosynthesis light response curve	μmol CO_2_ μmol^–1^ photon
θ_c_	The convexity of the non-rectangular hyperbolic curve of the canopy photosynthesis light response curve	Dimensionless

## Abbreviations

APAR: Absorbed PAR
A-Q curve: Light response curve of leaf photosynthesis
A_*cr*_-Q curve: Light response curve of canopy photosynthesis
IPAR: Intercepted PAR
PAR: Photosynthetically active radiation (from 400 to 700 nm wavelength), which is expressed as photosynthetic photon flux density (PPFD)
Abs_*canopy*_: Canopy absorption coefficient
multi-CAPTS: multi-chamber canopy photosynthesis and transpiration systems
DAS: Days after sowing.
