# Supplementary material for: Diurnal and Seasonal Variations of Photosynthetic Energy Conversion Efficiency of Field Grown Wheat
Source: Front Plant Sci. 2022 Feb 25;13:817654. doi: 10.3389/fpls.2022.817654 (PMC8914475; doi:10.3389/fpls.2022.817654)
Supplement: Supplementary file 1 [file Data_Sheet_1.pdf]

Qingfeng Song[1], Jeroen Van Rie[2], Bart Den Boer[2], Alexander Galle[2], Honglong Zhao[1], Tiangen Chang[1], Zhonghu He[3] and Xin-Guang Zhu[1,*]

[1]National Key Laboratory of Plant Molecular Genetics, CAS Center for Excellence in Molecular Plant Sciences, Institute of Plant Physiology and Ecology, Chinese Academy of Sciences, Shanghai 200032, China.
[2]BASF Belgium Coordination Center – Innovation Center Gent, Technologiepark-Zwijnaarde 101, 9052 Gent, Belgium
[3]Institute of Crop Sciences, Chinese Academy of Agricultural Sciences

Diurnal and Seasonal Variations of Photosynthetic Energy Conversion Efficiency of Field Grown Wheat

SUPPLEMENTARY METHODS

Calculation of the net CO_2 flux (Fc)

$$F_c = \frac{dc}{dt} \cdot \frac{P \cdot V}{R \cdot S \cdot T} \qquad \text{(Eqn S1)}$$

Where P (unit: kPa) is air pressure in the canopy chamber, V (unit: m^3) is the volume of air in the chamber, S (unit: m^2) is the ground area covered by the canopy, T (unit: K) is the air temperature and R (8.314×10^{-3} m^3 kPa mol^{-1} K^{-1}) is universal gas constant.

Determination of canopy light absorption coefficient

The PAR incident on a canopy (I) was measured with a quantum sensor (LI-191, LiCOR, Lincoln, Nebraska USA) placed horizontally above the canopy. The PAR reflected by the canopy (I_r) was measured with a quantum sensor placed above a canopy facing downward, and the PAR transmitted through the canopy (I_t) was measured with a quantum sensor placed at the bottom of the canopy facing upward. The canopy light absorption coefficient (α) was calculated by (Eqn S2). The PAR absorbed by a canopy (I_a) was calculated by the incident PAR multiplied by α (Eqn S3). The canopy light absorption coefficient around noon was determined and used to represent α for the whole day (Earl and Davis, 2003) because there is a linear relationship (1:1) between solar noon PAR interception and daily PAR interception measured in previous

studies (Daughtry et al., 1992; Earl and Davis, 2003).

$$\alpha = \frac{I - I_r - I_t}{I} \quad \text{(Eqn S2)}$$

$$I_a = I \times \alpha \quad \text{(Eqn S3)}$$

Above ground biomass measurement

At each wheat developmental stage, the above ground biomass (BM) from one m^2 ground area (about 100 plants) was harvested from the center of an experimental plot. The dry weight was measured after drying the biomass in an oven at 110 ºC for one hour followed by 80 ºC for 3 days.

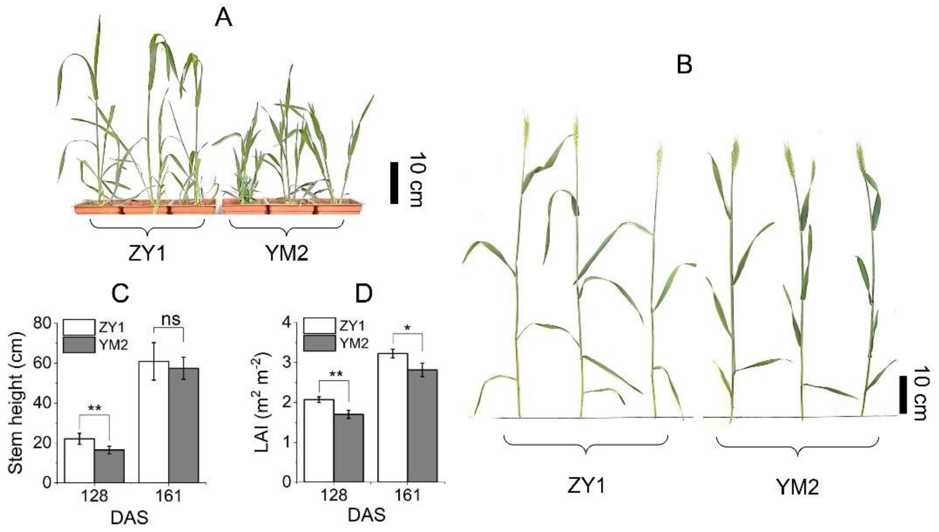

Figure S1 Canopy architectures of the two wheat cultivars, ZY1 and YM2, on the 128th DAS during the booting stage (A) and on the 161st DAS during the early grain filling stage (B). Stem height (C) and leaf area index (D) at the booting stage and the early grain filling stage. Data are shown as mean ± sd (n=6). ** represent $P < 0.01$ and * represent $P < 0.1$ determined by the Student's *t* test.

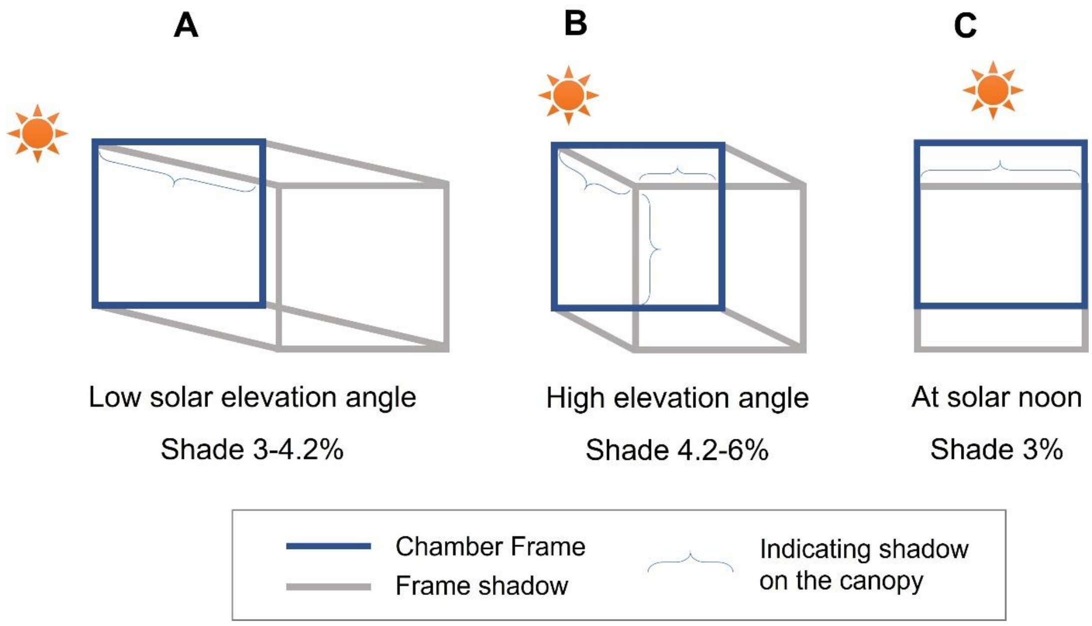

Figure S2, Diagram showing the proportions of shaded area caused by chamber frame to the canopy. The diagram shows the top view of the chamber and shadows for the frame on the ground at three different solar elevation angles. Only those shadows indicated by the brackets fall within the chamber, which is about 3-6% of the total ground area covered by the chamber. The width of the frame is 3cm and the ground area covered by the chamber is 100cm * 100cm.

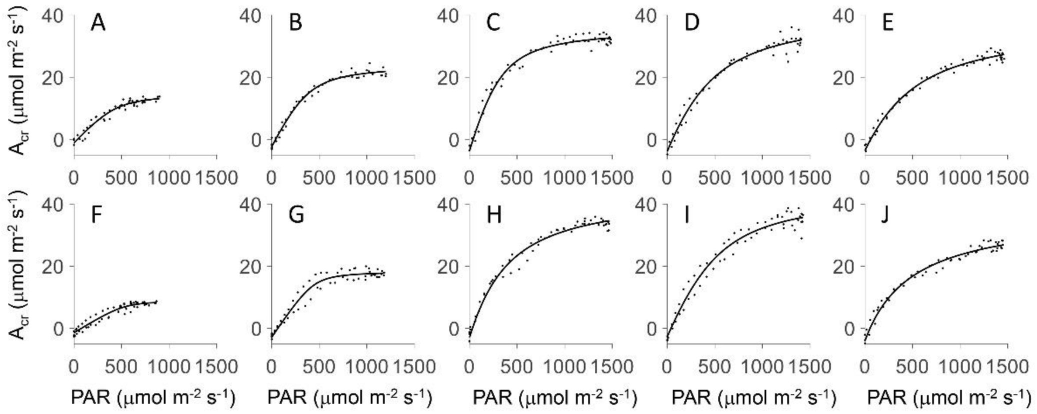

Figure S3 The response of whole plant CO_2 flux (A_{cr}) under different photosynthetically active radiation per ground area (PAR) at different developmental stages for field-grown wheat. A&F: the tillering stage; B&G: the booting stage; C&H: the heading stage; D & I: early grain filling stage; E & J: the late grain filling stage. A, B, C, D, E are for ZY1, while F, G, H, I, J are for YM2. The data points represent measured data with multi-CAPTS and the black curves are the regression lines of the measured data with a non-rectangular hyperbola equation (Eqn. 5).

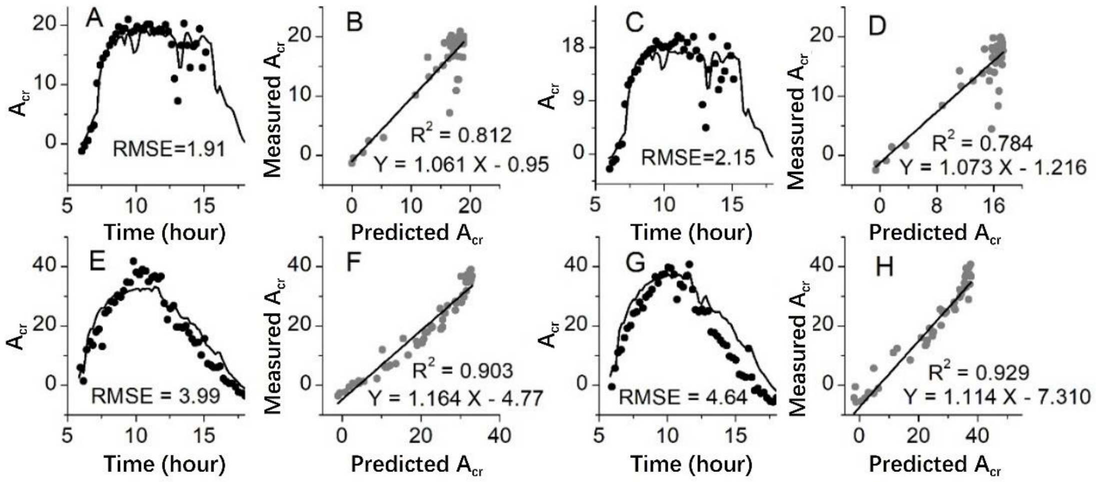

Figure S4 Comparison of the model predicted A_{cr} and measured A_{cr} at different stages for both ZY1 and YM2. A, B: ZY1 on the 28th of March; C, D: YM2 on the 28th of March; E, F: ZY1 on the 1st of May; G, H: YM2 on the 1st of May. In panel A, C, E and G , black dots represent the measured A_c and the line represents the predicted A_c, the RMSE (Root Mean Squared Error) between prediction and measurement was also shown on each panel. Panel B, D, F and H show the comparison between predicted A_{cr} and measured A_{cr} with equations and R^2 of linear regression.

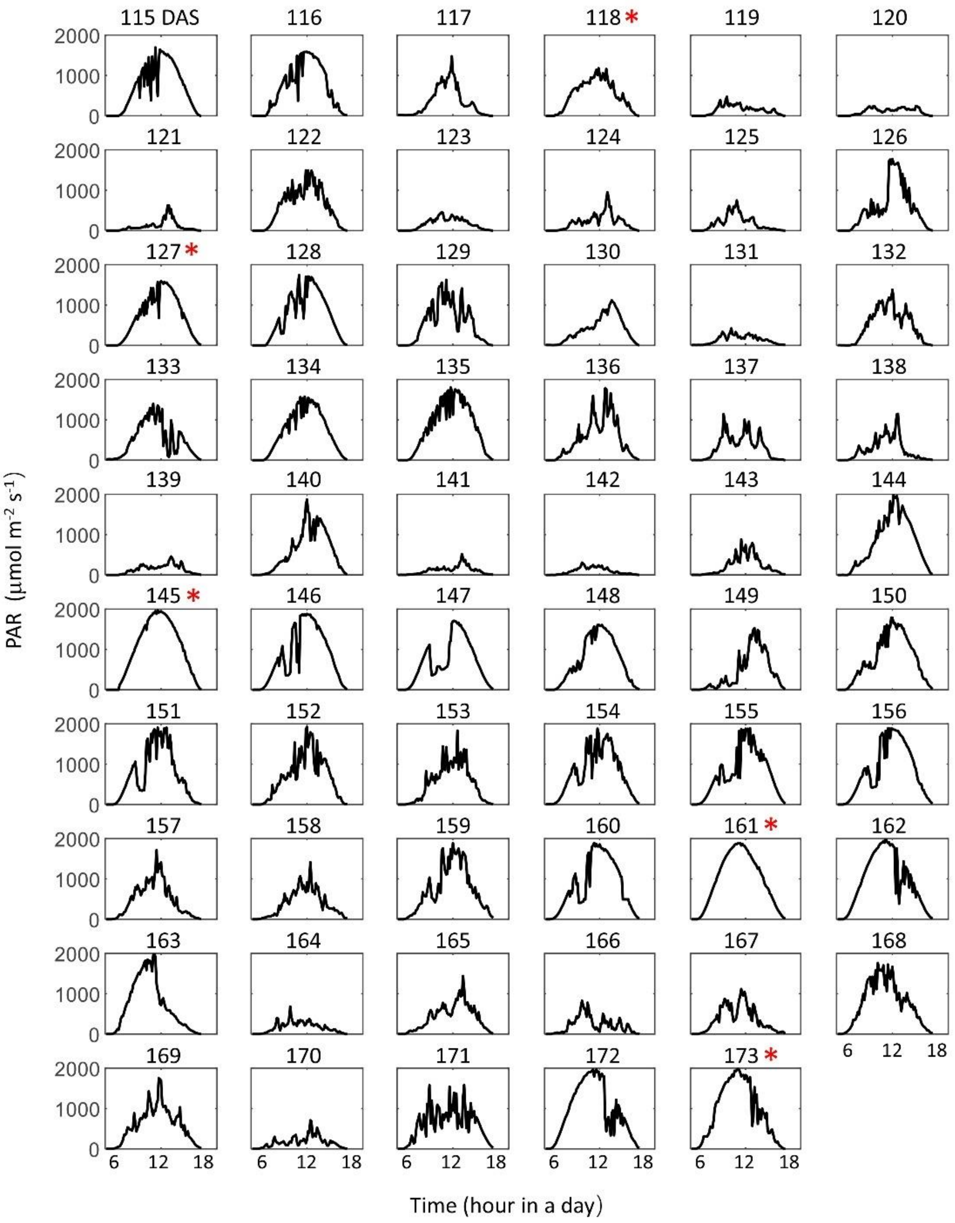

Figure S5. The photosynthetically active radiation (PAR) from the 14th of March (115 days after sowing, DAS) to the 11th of May (173 DAS) in 2017. The data were measured and recorded by a weather station in the Songjiang experiment field in Shanghai, China. The ambient PAR was recorded with a 10- minute interval. The days labeled with red asterisk were used to measured canopy gas exchange.

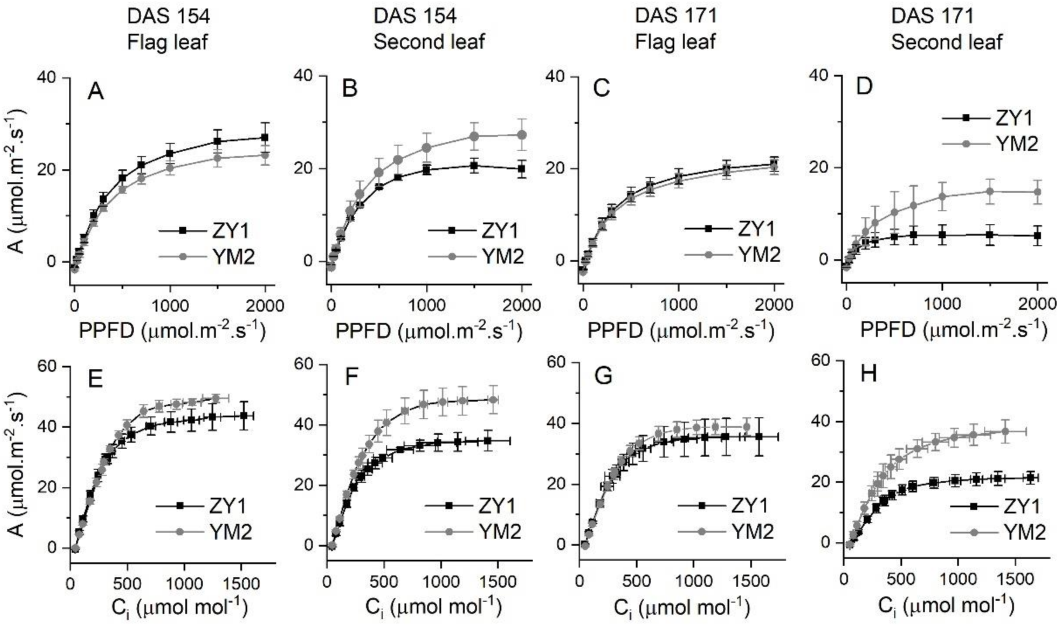

Figure S6, Light response curves (A-Q curve) and CO_2 response curves (A-Ci curve) of leaf photosynthetic CO_2 uptake rates for the top two leaves (FL: flag leaf, L2: the second leaf from the top) measured at the grain filling stage (from the 154th and the 171st DAS). On the 154th DAS, the A of the flag leaf for YM2 was lower than that for ZY1, but A of the second leaf for YM2 was higher than ZY1; on the 171st DAS, for both flag leaf and the second leaf, the leaf photosynthesis rates of YM2 were higher than those of ZY1.

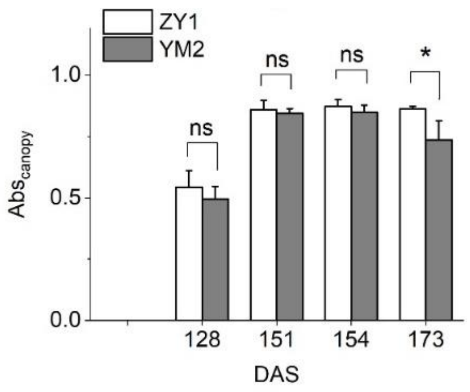

Figure S7 Canopy absorbance (Abs_{canopy}) at different growth stages. Data shown are mean±sd (n=3). We fitted a quadratic equation between Abs_{canopy} and DAS as $Y = -2.97*10^{-4} X^2 + 0.0965 X – 6.94$ for ZY1 and $Y = -4.40*10^{-4} X^2 + 0.138 X – 9.92$ for YM2. * represents $P< 0.1$ based on the Student's *t* test and ns represent non-significant difference.

Table S1

To test the effects of scatter light on canopy photosynthesis, we used two kinds of PVC, both having a transmittance of 0.9. However, one of the PVC has 50% of the transmitted light converted into scattering light (PVCs), and the second PVC did not alter the ratio of the scattering light to direct light (PVC). Canopy photosynthesis measurement was performed on two days: on the first day, chambers were used without covering, which were used as control; on the second day, the chambers were covered by PVC and PVCs (Plot 1-3: covered with PVC, plot 4-6: covered with PVCs) and the relative change of A_{cr} comparing to the control was calculated. Canopy photosynthesis of plots covered with PVCs was increased by 6.3% comparing to the plots covered with PVC.

Plots	Day1	Day2	Relative Change of A_{cr}	Averaged value of the relative change of A_{cr}
**Plot1**	0.714 (NC)	0.664 (**PVC**)	0.930	
**Plot2**	0.972 (NC)	0.951 (**PVC**)	0.978	0.945
**Plot3**	1.011 (NC)	0.937 (**PVC**)	0.927	
**Plot4**	0.753 (NC)	0.744 (**PVCs**)	0.989	
**Plot5**	1.138 (NC)	1.171 (**PVCs**)	1.029	1.008
**Plot6**	1.192 (NC)	1.198 (**PVCs**)	1.005	

Table S2

To test the impact of solar elevation angle to the transmittance of canopy chamber, we measured the transmittance of the PC film. Relative data were shown for three replicates and the averaged value.

Incident angle	Transmittance of PC (relative value)			average
**0 degree**	1.000	1.000	1.000	1.000
**10 degrees**	0.997	0.997	0.998	0.997
**20 degrees**	0.995	0.996	0.995	0.995
**30 degrees**	0.985	0.993	0.993	0.990
**40 degrees**	0.979	0.987	0.989	0.985
**50 degrees**	0.968	0.984	0.976	0.976
**60 degrees**	0.961	0.978	0.969	0.970
**70 degrees**	0.943	0.975	0.949	0.956

Table S4

To evaluate the impacts of different weather on accuracy of A_{cr} prediction, we built A_{cr}-Q models with data from different days including both sunny days and cloudy days at different stages. The RMSEs (unit: μmol m^{-2} s^{-1}) of predicting A_{cr} of three days (day1, day2 and day3 represent three days at one stage) with the A_{cr}-Q model at the same stage were calculated. The average PAR (unit: μmol m^{-2} s^{-1}) was calculated to show the ambient light conditions of the days used for developing A_{cr}-Q model. The stage 1, 2, 3 and 4 represent the tillering, booting, heading and early grain filling stages.

Stage	Date for building A_{cr}-Q model		Cultivar	Plot	Average PAR	RMSE of A_{cr}-Q model prediction for three days at the same stage		
	Month	Day				day1	day2	day3
1	3	16	ZY1	1	393	0.89	1.31	1.39
1	3	17	ZY1	1	538	1.14	1.07	1.59
1	3	18	ZY1	1	161	2.42	2.41	1.30
2	3	26	ZY1	1	644	1.52	2.66	2.99
2	3	27	ZY1	1	725	2.14	2.08	2.57
2	3	28	ZY1	1	635	2.48	2.39	2.17
3	4	13	ZY1	1	847	2.79	3.31	3.93
3	4	14	ZY1	1	825	3.14	2.99	2.99
3	4	16	ZY1	1	653	4.39	3.89	1.78
4	4	29	ZY1	1	831	2.08	2.27	2.77
4	4	30	ZY1	1	869	2.21	2.15	2.23
4	5	1	ZY1	1	643	2.86	2.50	1.82
1	3	16	ZY1	2	386	1.11	1.14	1.71
1	3	17	ZY1	2	536	1.13	1.11	1.74
1	3	18	ZY1	2	159	1.95	1.92	0.87
2	3	26	ZY1	2	638	1.22	1.61	2.21
2	3	27	ZY1	2	727	1.50	1.29	2.17
2	3	28	ZY1	2	612	1.90	1.92	1.64
3	4	13	ZY1	2	846	1.88	2.48	4.34
3	4	14	ZY1	2	822	2.38	1.99	3.23
3	4	16	ZY1	2	653	4.04	3.15	2.12
4	4	29	ZY1	2	847	1.32	1.55	2.59
4	4	30	ZY1	2	868	1.50	1.38	2.50
4	5	1	ZY1	2	636	2.25	2.20	1.69
1	3	16	ZY1	3	408	0.61	1.34	1.25
1	3	17	ZY1	3	540	1.01	1.03	1.49
1	3	18	ZY1	3	160	2.23	2.24	1.05
2	3	26	ZY1	3	639	1.00	2.21	3.64
2	3	27	ZY1	3	741	1.87	1.43	2.53
2	3	28	ZY1	3	610	3.27	2.22	1.76
3	4	13	ZY1	3	842	1.51	2.13	4.61

3	4	14	ZY1	3	824	1.98	1.77	3.67
3	4	16	ZY1	3	660	4.31	3.44	1.75
4	4	29	ZY1	3	845	1.22	1.37	2.70
4	4	30	ZY1	3	852	1.30	1.30	3.09
4	5	1	ZY1	3	633	2.41	2.66	1.41
1	3	16	YM2	1	348	0.67	1.29	0.82
1	3	17	YM2	1	517	0.85	1.15	0.77
1	3	18	YM2	1	141	1.03	1.62	0.66
2	3	26	YM2	1	732	1.34	2.49	2.54
2	3	27	YM2	1	773	1.91	1.97	2.34
2	3	28	YM2	1	649	2.03	2.48	1.94
3	4	13	YM2	1	865	2.07	2.58	3.90
3	4	14	YM2	1	818	2.43	2.25	3.09
3	4	16	YM2	1	684	4.23	3.62	1.75
4	4	29	YM2	1	850	2.33	2.37	3.88
4	4	30	YM2	1	859	2.62	2.02	2.84
4	5	1	YM2	1	647	3.92	3.15	1.88
1	3	16	YM2	2	355	0.68	1.01	0.88
1	3	17	YM2	2	515	0.85	0.90	0.71
1	3	18	YM2	2	135	1.60	2.14	0.64
2	3	26	YM2	2	724	1.51	1.99	2.87
2	3	27	YM2	2	773	2.06	1.38	2.20
2	3	28	YM2	2	627	2.63	1.88	1.80
3	4	13	YM2	2	884	1.86	2.13	3.48
3	4	14	YM2	2	818	2.11	1.92	2.69
3	4	16	YM2	2	670	3.73	3.09	1.27
4	4	29	YM2	2	847	1.67	1.84	3.51
4	4	30	YM2	2	864	1.85	1.68	2.99
4	5	1	YM2	2	637	3.16	2.70	1.93
2	3	26	YM2	3	727	0.99	2.70	3.26
2	3	27	YM2	3	766	2.16	1.92	2.04
2	3	28	YM2	3	610	2.82	2.21	1.73
3	4	13	YM2	3	869	1.45	1.66	3.23
3	4	14	YM2	3	817	1.63	1.55	2.76
3	4	16	YM2	3	671	3.35	2.90	1.35
4	4	29	YM2	3	864	1.04	1.47	3.34
4	4	30	YM2	3	866	1.35	1.23	2.86
4	5	1	YM2	3	638	3.37	2.97	1.23

Table S5

The influences of parameters derived from the A_{cr}-Q curve on the light use efficiency (LUE). The sensitivity analysis was performed by increasing or decreasing 10% of values for P_{cmax}, Φ_c and θ. The stage 2, 3 and 4 represent the booting, heading and early grain filling stages.

cultivar	stage	The change of the predicted LUE (%) by changing the A_{cr}-Q parameters					
		P_{cmax}+10%	P_{cmax}-10%	Φ_c+10%	Φ_c-10%	θ+10%	θ-10%
ZY1	2	9.19%	-9.75%	3.90%	-4.46%	14.21%	-8.36%
ZY1	3	6.57%	-7.06%	2.92%	-3.65%	0.73%	-0.97%
ZY1	4	5.47%	-5.78%	4.56%	-4.56%	0.00%	0.00%
YM2	2	9.58%	-9.90%	3.19%	-3.51%	9.58%	-8.63%
YM2	3	8.43%	-8.99%	3.65%	-3.93%	3.37%	-2.81%
YM2	4	8.53%	-9.69%	4.65%	-5.81%	0.00%	-0.39%

Table S6

To evaluate the influence of A_{cr}-Q parameters on the energy conversion efficiency (ε_c). The sensitivity analysis was performed by increasing or decreasing 10% of values for P_{cmax}, Φ_c and θ. The stage 2, 3 and 4 represent the booting, heading and early grain filling stages.

cultivar	stage	The change of the predicted ε_c by changing the A_{cr}-Q parameters					
		P_{cmax}+10%	P_{cmax}-10%	Φ_c+10%	Φ_c-10%	θ+10%	θ-10%
ZY1	1	0.08%	-0.11%	0.09%	-0.11%	0.20%	-0.21%
ZY1	2	0.18%	-0.19%	0.08%	-0.09%	0.29%	-0.16%
ZY1	3	0.23%	-0.25%	0.10%	-0.13%	0.03%	-0.03%
ZY1	4	0.17%	-0.18%	0.14%	-0.15%	0.00%	0.00%
YM2	1	0.08%	-0.09%	0.05%	-0.05%	0.16%	-0.11%
YM2	2	0.17%	-0.17%	0.05%	-0.05%	0.16%	-0.14%
YM2	3	0.27%	-0.29%	0.11%	-0.13%	0.11%	-0.10%
YM2	4	0.22%	-0.23%	0.12%	-0.13%	0.01%	0.00%

Reference:

Daughtry, C.S.T., Gallo, K.P., Goward, S.N., Prince, S.D., Kustas, W.P., 1992. Spectral estimates of absorbed radiation and phytomass production in corn and soybean canopies. Remote Sens. Environ. 39, 141–152. https://doi.org/10.1016/0034-4257(92)90132-4

Earl, H.J., Davis, R.F., 2003. Effect of drought stress on leaf and whole canopy radiation use efficiency and yield of maize. Agron. J. 95, 688–696. https://doi.org/10.2134/agronj2003.0688
